# PB.5. Accuracy of axillary nodal staging on MRI of the breasts: correlation with ultrasound of the axilla and histopathology findings

**DOI:** 10.1186/bcr3739

**Published:** 2014-11-03

**Authors:** M Wilson, A Maxwell, S Gadde, E Hurley, M Bydder, E Harkness, M Ewins, S Astley, Y Lim

**Affiliations:** 1 The Nightingale Centre and Genesis Prevention Centre, University Hospital of South Manchester, Manchester, UK; 2 Centre for Imaging Sciences, University of Manchester, UK; 3 University of Manchester Medical School, Manchester, UK

## Introduction

Ultrasound (US) is routinely used for preoperative staging of the axilla in women with breast cancer but this has limitations with variable sensitivities published in the literature. Magnetic resonance imaging (MRI) of the breasts is frequently performed for preoperative staging of the breasts, and the axilla can be visualised on these scans. The aim of this study is to assess the accuracy of axillary nodal staging on MRI of the breasts.

## Methods

A 4-year retrospective study was performed that included 205 women with breast carcinoma who underwent preoperative staging MRI of the breasts. Two consultant radiologists reviewed the MRI. The nodes were assessed for loss of fatty hilum, eccentric cortical thickness and maximal short axis diameter >10 mm. The findings were recorded on a proforma and subsequently correlated with the US findings and surgical histology results.

## Results

For the 205 cases, the sensitivity (95% CI) was 55% (48 to 62%) for MRI and 49% (42 to 56%) for US. The specificity was 88% (84 to 92%) for MRI and 98% (96 to 100%) for US. The combination of MRI and US resulted in an increase in sensitivity to 79% (73 to 85%) with a specificity of 84% (79 to 89%).

## Conclusion

There was no statistically significant difference in the sensitivity and specificity of preoperative axillary nodal staging between MRI and US. The combination of MRI and US has been shown to have significantly higher sensitivity. Careful evaluation of the axilla on MRI to identify the cases for second-look US may increase the accuracy of preoperative staging of the axilla.

